# Human longevity: Genetics or Lifestyle? It takes two to tango

**DOI:** 10.1186/s12979-016-0066-z

**Published:** 2016-04-05

**Authors:** Giuseppe Passarino, Francesco De Rango, Alberto Montesanto

**Affiliations:** Department of Biology, Ecology and Earth Science, University of Calabria, 87036 Rende, Italy

**Keywords:** Human longevity, Genetics of aging, Calorie restriction

## Abstract

Healthy aging and longevity in humans are modulated by a lucky combination of genetic and non-genetic factors. Family studies demonstrated that about 25 % of the variation in human longevity is due to genetic factors. The search for genetic and molecular basis of aging has led to the identification of genes correlated with the maintenance of the cell and of its basic metabolism as the main genetic factors affecting the individual variation of the aging phenotype. In addition, studies on calorie restriction and on the variability of genes associated with nutrient-sensing signaling, have shown that ipocaloric diet and/or a genetically efficient metabolism of nutrients, can modulate lifespan by promoting an efficient maintenance of the cell and of the organism. Recently, epigenetic studies have shown that epigenetic modifications, modulated by both genetic background and lifestyle, are very sensitive to the aging process and can either be a biomarker of the quality of aging or influence the rate and the quality of aging.

On the whole, current studies are showing that interventions modulating the interaction between genetic background and environment is essential to determine the individual chance to attain longevity.

## Background

The research on aging, and in particular the search for the determinants of successful aging and longevity, has been continuously growing in the last decades also due to the social and medical burden correlated to the continuous increase of lifespan in western countries and the consequent grow of the elderly population. One of the main questions in this field is the correlation between the genetic background and lifestyle in determining the individual chance of a delayed aging (possibly without age-related diseases and disabilities) and longevity. The results obtained by biogerontologists in these years, which highlighted most of the biological and biochemical mechanisms involved in the aging process, allowed to better understand such correlation. This has brought to elaborate important strategies focused on possible interventions to improve lifestyle in order to increase the chance to attain longevity by modulating the basic molecular mechanisms of aging.

### The genetics of aging

Before the 1990ies it was largely spread the idea that aging is ineluctable and that genetics does not control it. It was important, in this view, the idea that aging occurs after reproduction, and then there is no need, but also no opportunity, for selection to act on genes that are expressed during this late period of life [1].

The researcher who pioneered the genetics of aging and longevity was Tom Johnson, who studied groups of *C. elegans* where he was able to separate long living individuals from short living subjects. The analysis of hybrids obtained from different strains of *C. elegans,* allowed to estimate that the heritability of life-span was between 20 and 50 % [2, 3]. Subsequently, he started the analysis of different mutants and, with M. Klass, found a number of mutants with longer lifespan. Subsequently, Tom Johnson found out that most of the mutants with long lifespan had mutations in the *age1* gene [4]. This gene turned out to be the catalytic subunit of class-I phosphatidylinositol 3-kinase (PI3K).

The studies of Johnson clearly demonstrated that genetic variability could indeed affect lifespan. This triggered many studies in model organisms in order to disentangle the different biochemical pathways which could affect lifespan, and to highlight the genes coding for the proteins involved in such pathways. In particular, yeast, *C. elegans*, drosophila and mice were analyzed and this highlighted numerous genes which could affect lifespan if mutated (for an updated list of these genes see http://genomics.senescence.info/genes/models.html). Most of these genes are related to the maintenance of the integrity of the cell (especially the integrity of DNA). In *C. elegans*, however, some of the main genes which have been found to modulate lifespan (*daf2*, *daf16*) are related to the ability to enter the *dauer* status [5, 6], that is a quiescent status (usually entered in case of nutrient deprivation) with a minimum energy expense, which causes an arrest of the reproduction process and allows the organism to live longer “expecting” for the availability of nutrients. This suggested that longevity can be attained by means of an efficient maintenance of the cell but also by diverting resources from reproduction to self maintenance, in line with previous findings that dietary restriction can extend lifespan. After the characterization of these genes in *C. elegans*, it was found that in mice the ortholog of *daf16* (FOXO) could affect lifespan. In mammals, FOXO is correlated to the Insulin/IGF1 axis which is stimulated by nutrient availability and, through FOXO, promotes protein synthesis [7–11].

It is of note that some Authors suggested these molecular mechanisms modulating lifespan could be due to a pleiotropic effect of genes which have evolved for different purposes (such as the genes in the IGF-1 pathway which have evolved to face presence/absence of nutrients) but can, ultimately affect lifespan; others proposed that some genes may have evolved to program aging and avoid “immortality”, as this would hamper the continuous substitution of old subjects with new, younger, ones [12, 13].

It was obviously inevitable that the research of the genetic basis of longevity turned to human beings and investigated whether the common genetic variability of human populations could affect inter individual differences in lifespan but also whether the genes found to prolong lifespan in model organisms, on turn, were correlated to human lifespan.

As to the first question (does common genetic variability affect lifespan, and in particular does it affect longevity?), this has been studied by two approaches. The first one was the reconstruction of the sibships of long-lived subjects [14, 15] and the comparison of their survival curves with those of the birth cohorts born in the same geographical area. This approach demonstrated that brothers and sisters of the long-lived subjects had a clear survival advantage (at any age) with respect to the general population. The second approach, with intrafamily controls, was started in order to distinguish the genetic from the “familiar” effect. Montesanto et al. [15] compared the survival function of brothers of centenarians with those estimated for their brothers in law, that is with the men who married their sisters; these men were supposed to share with the brothers of the long lived subjects the familiar environment. By using this second approach, it has been found that the survival advantage of siblings of long-lived subjects was not completely shared by their brothers in law, despite they shared the same environment for most of their life. This suggested that beyond the family environment, there are genetic factors influencing survival and, consequently, lifespan. Interestingly, in this study, the survival curve of the sisters of long-lived subjects did not differ from the one of sisters in law, suggesting that the genetic component does explain lifespan in men more than in women. The genetic component of lifespan in humans has also been analyzed by comparing the age of death of monozygotic and dizygotic twins. This has allowed to estimate that about 25 % of the variation in human longevity can be due to genetic factors and indicated that this component is higher at older ages and is more important in males than in females [16–18].

In parallel to these studies, many researches have been carried out to search the genetic variants responsible of modulating human longevity. Most of them were carried out by a case/control approach, by comparing the frequency of specific polymorphisms in long-lived subjects and in younger geographically matched controls. The rationale of this study design is that as the population ages, alleles favorable for survival will be present at higher frequency among long-living people, while unfavorable alleles will be eliminated [19–21]. The candidate genes analyzed by this approach were either genes involved in age-related diseases (such as APOE, which had been observed to be involved in the predisposition to Alzheimer Disease and other age-related cognitive impairments), or genes implicated in pathways related to longevity in studies with model organisms (IGF-1, FOXO, Sirtuins) [22–25]. This study design has indeed led to find numerous polymorphic genes the variability of which affects longevity. However, each of these polymorphisms turned out to explain only a very small fraction of the longevity variability. Indeed high-throughput Genome-wide analyses, which have recently been carried out have identified many genes positively associated with longevity but only a very few ones could hold multiple test significance and successfully replicated in different studies and across different populations [26–29]. Population stratification and inadequate sample sizes are among the main plausible explanations [30]. The adoption of innovative study design and the development of new statistical and computational tools for effective processing of genetic data arising from high-throughput DNA technologies will help to better understand the complex genetic architecture underlying human longevity [31, 32].

A new way of looking at the genetic data has been proposed by Raule et al. [33] who analyzed the complete sequences of mitochondrial DNA from long-lived subjects coming from different areas of Europe. The availability of complete sequences allowed to evaluate for the first time the cumulative effects of specific, concomitant mitochondrial DNA (mtDNA) mutations, including those that *per se* have a low, or very low, impact. The analysis indicated that the presence of single mutations on mtDNA complex I may be beneficial for longevity, while the co-occurrence of mutations on both complexes I and III or on both I and V might lower the individual’s chances for longevity. Previous analyses on single mutations falling on complex I (either specific mutations or mutations defining groups of haplotypes) had given contrasting results, showing association with longevity in some cases but not in others. It is likely that positive results were obtained in populations were mutations on complex I were not associated with mutations on complex III or V, while negative results were obtained in populations with high prevalence of mtDNA haplotypes carrying mutations on complex I in association with mutations in complex III and V. This approach confirmed that most of the genetic variants have a very limited effect on longevity, and that only their cumulative effect can give a consistent appreciable effect and suggests that a limit of previous analyses has been to search for single mutations instead of cumulative effects. On the other hand, it is very difficult to think of using such approach, which has been successful for mitochondrial DNA, on genomic DNA unless small fractions (or specific regions harboring genes involved in relevant pathways) are analyzed.

On the whole, the genetic association studies suggested that, also in humans, mutations in genes correlated with the maintenance of the cell and of its basic metabolism are essential in modulating lifespan. Indeed, genes involved in DNA repair [34], telomere conservation [35–37], heat shock response [38, 39], and the management of free radicals’ levels [33, 40] were found to contribute to longevity or, in case of reduced functionality, to accelerated senescence (cellular aging) and the consequent organism aging. In addition, as suggested by the studies in mice, the pathways involved in nutrient-sensing signaling and in regulating transcription, such as IGF-1/insulin axis [41] and TOR (target of rapamycin) [42] showed to be involved in modulating human longevity. Besides these genes involved in cellular maintenance/metabolism and senescence, concurrent efforts, especially from clinical studies, also showed that genes implicated in important organismal process may have a strong impact on aging and longevity. For instance genes involved in lipoprotein metabolism (especially APOE), cardiovascular homeostasis, immunity, and inflammation have been found to play an important role in aging, age-related disorders, and organism longevity [43–46].

### Human longevity and life style

Life expectancy at birth has been increasing for most of the last century in western societies, thanks to the continuous amelioration of medical assistance, to the improvement of the environment (in particular clean, safe water and food), and to the improvement of nutrients. For instance, in Italy life expectancy went from 29 years in 1861 to 82 in 2011 (Table 1 reports the evolution of this data in women and men). Similarly, the extreme longevity has been growing in these years. Indeed, the number of centenarians (still in Italy) remarkably increased from 165 in 1951 to more than 15000 in 2011. These results have been attained first by a dramatic reduction of infectious diseases, which, on turn, has dramatically reduced infantile mortality, but also mortality in adult age. In fact, in 2011 less than 10 % of deaths occurred in subjects under 60 years of age, while the corresponding figures were 74 % in 1872, 56 % in 1901 and 25 % in 1951. However, in the last decades, the continuous extension of lifespan was mainly due to the improvement of medical assistance with respect to age-related diseases, especially Cardiovascular Diseases and Cancer, which allowed to increase lifespan of 5 years in the last 2 decades and of 2 years in the last 10 years (data from www.mortality.org and www.istat.it).

**Table 1:** Evolution of lifespan expectancy in Italy from 1861

Year	Male	Female	Total
1861	28	29	29
1871	30	31	30
1881	35	35	35
1891	38	39	38
1901	43	43	43
1911	46	46	46
1921	48	50	49
1931	53	56	55
1941	55	58	56
1951	63	67	65
1961	67	72	69
1971	69	75	72
1981	71	78	75
1991	74	80	77
2001	77	83	80
2011	79	84	82

These data clearly show that environmental factors have a very strong impact on lifespan and on longevity in humans. However, the extension of lifespan that there has been in the last decades have not been accompanied by a similar extension of healthy lifespan. Indeed, in most cases this lifespan extension is due to the chronicit of the age-related diseases. This has brought the community of biogerontologists to study interventions, possibly modulated on the knowledge emerged from the studies on the genetic and biomolecular basis of longevity, to extend not only lifespan but also healthy lifespan, or, with a new word, “healthspan”. In fact, model organisms with mutations that extend lifespan have a healthy life also when they are old. This suggested that health span extension could be attained by targeting (stimulating or silencing) the genes, which had been highlighted to be involved in life extension in both model organisms and humans [47]. In support of this hypothesis, it has been reported that dietary restricted mice, which live much longer and show a very delayed aging phenotype than mice fed *at libitum*, at old age have an expression pattern very different from mice of the same age for a number of genes correlated with life extension, such as those related to DNA repair, stress response, immune response and others [48, 49]. Thus, dietary restriction can trigger a molecular-genetic response which postpones aging and age-related phenotypes. This has brought to search for drugs or interventions which may act on these mechanisms without the side effects of calorie restriction. Among the most important interventions which have been considered in this context, we may name the protein restriction, the use of drugs targeting different genes of IGF-1 axis or of the FOXO/TOR pathway [47]. In addition, these studies have allowed to reconsider previous data on some areas characterized by exceptional longevity (such as Okinawa, Sardinia and Calabria) which are characterized by traditional ipoproteic diets, such as the “Mediterranean diet” [50–53]. In these cases, then, the environment, that is the traditional diet, has allowed to stimulate the molecular mechanisms which can increase life span.

Among the several changes that occur with the aging process, in the last decade Epigenomics has attracted the interest of many researchers. This was mainly due to the fact that epigenetic modifications summarizing, at least in part, the interaction between the individual genetic background and lifestyle characteristics, should be potentially able to capture part of the unexplained susceptibility observed today for complex diseases (the so-called missing heritability problem).

Starting from the pioneeristic observations that epigenetic modifications affect not only the aging process but also its quality (successful aging) [54], EpiGenome-Wide Association Studies identified hundreds of sites spread along the entire genome in which methylation levels change between oldest old and younger subjects. In particular, Horwat and co-workers, on the basis of the methylation levels of 353 CpG units, formulated a mathematical model, the so-called epigenetic clock, that showed some important properties [55]. First, it was able predict the chronological age of a subject starting from the methylation level of several cells and tissues of his body. Second, it represents one of the most accurate biomarker of age (also superior to the estimates obtained from the telomere length). Third, using methylation levels of blood and brain tissues from subjects affected by Down syndrome, it showed that an accelerated aging occur in such a syndrome [56]. Fourth, it was able to predict all-cause mortality also after adjusting for traditional risk factors [57]. Finally, when it was used to estimate the biological age of several tissues from supercentenarians, it has been demonstrated that brain and muscle represent the youngest tissues of these exceptional individuals [58].

However, even if the cause-effect relationship between methylation process and aging is still not clear, the potential applications of this discovery are very wide, ranging from detailed monitoring of changes occurring with age within individual systems or organs (muscle, brain, etc.) to forensic purposes. For this and several other reasons, future advances in this field could help the understanding of the complex physiology of aging, lifespan and age-associated diseases.

## Conclusions

On the whole, although the common variability accounts for only 25 % of human lifespan variability, the knowledge of the genetic basis modulating longevity may give significant hints on modulating lifestyle in order to attain longevity and extend healthspan. That is, a few subjects can attain longevity because a lucky combination of polymorphisms which allow them to have an efficient metabolism or an efficient response to different stress. Most of the others can attain a similar result by targeting the same pathways with appropriate life style or interventions. In this context, the importance of epigenetic factors, both as biomarkers of aging and target of interventions will certainly grow in the forthcoming future.

## Abbreviations

APOE: apolipoprotein E
FOXO: forkhead box O
IGF-1: insulin-like growth factor 1
mtDNA: mitochondrial DNA
PI3K: phosphatidylinositol-3-kinase
TOR: target of rapamycin
